# Blastic Plasmacytoid Dendritic Cell Neoplasm with Leukemic Component

**DOI:** 10.4274/tjh.galenos.2019.2018.0363

**Published:** 2019-08-02

**Authors:** Maria Jimenez Esteso

**Affiliations:** 1 Hospital Marina Baixa, Clinic of Hematology, Alicante, Spain; 2 Hospital General Universitari d’Alacant, Clinic of Hematology, Alicante, Spain

**Keywords:** Mast cells, Dendritic cells, Acute leukemia

A 72-year-old man was admitted to our hospital with a reddish skin tumor, which had appeared 3 weeks ago on his arm. The histopathological examination of a skin biopsy specimen led to the diagnosis of blastic plasmacytoid dendritic cell neoplasm (BPDCN) based on the World Health Organization’s 2008 classification (Figure 1). Physical examination revealed erythematous lesions on plaques, slightly indurated, on the back and arms.

Three months later, the patient came to the emergency department complaining of dizziness and feeling unwell. Physical examination revealed no significant findings. Complete blood count at this time showed a hemoglobin level of 10.2 g/dL, white blood cell count of 13.8x10^9^/L, and platelet count of 51x10^9^/L. Peripheral blood smear demonstrated 97% medium-sized blastoid cells with gray-blue cytoplasm, occasional cytoplasmic vacuoles, nucleoli, and some nuclear folds (Figures 2A and 2B). The bone marrow was packed (98% cellularity) with immature cells negative for myeloperoxidase, alpha-naphthyl butyrate esterase, and naphthol-ASD chloroacetate esterase staining and positive for periodic acid-Schiff (Figure 2C). Flow cytometry analysis showed blast cells expressing CD4/CD56/CD7/CD33/HLADR/dimCD45 with absence of CD34.

The diagnosis was BPDCN with involvement of peripheral blood. BPDCN is an aggressive hematologic malignancy originating from the precursors of plasmacytoid dendritic cells. It has a high frequency of cutaneous and bone marrow involvement and leukemic dissemination.

This neoplasm is a heterogeneous group of lymphoproliferative disorders, with different clinical, morphologic, and immunophenotypic features.

## Figures and Tables

**Figure 1:**
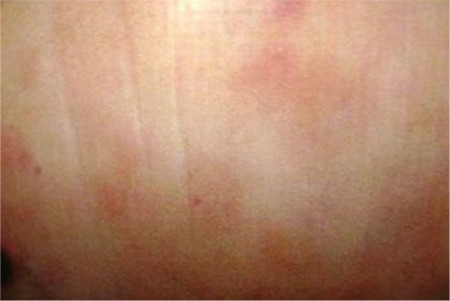
Cutaneous infiltration at diagnosis.

**Figure 2:**
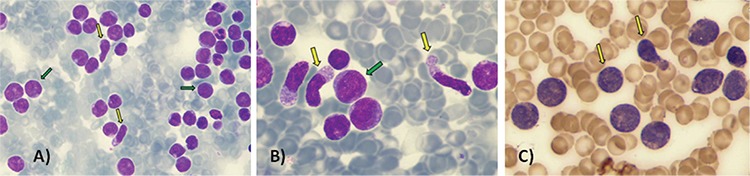
Peripheral blood smear demonstrated 97% medium-sized blastoid cells with gray-blue cytoplasm, occasional cytoplasmic vacuoles, nucleoli, and some nuclear folds (A, B). Bone marrow was packed (98% cellularity) with immature cells negative for myeloperoxidase, alpha-naphthyl butyrate esterase, and naphthol-ASD chloroacetate esterase staining and positive for periodic acid-Schiff (C). This neoplasm comprises a heterogeneous group of lymphoproliferative disorders with different clinical, morphologic, and immunophenotypic features.

